# Longitudinal study investigating the influence of COMT gene polymorphism on cortical thickness changes in Parkinson's disease over four years

**DOI:** 10.1038/s41598-024-60828-7

**Published:** 2024-04-30

**Authors:** Amin Tajerian

**Affiliations:** School of Medicine, Arak University of Medical Sciences, Arak, Iran

**Keywords:** COMT, Genetics, Neuroimaging, Cortical Thickness, PPMI, Parkinson’s disease, Mutation, Parkinson's disease, Neuroscience

## Abstract

Parkinson's disease (PD) is a progressive neurodegenerative disorder affecting over 3% of those over 65. It's caused by reduced dopaminergic neurons and Lewy bodies, leading to motor and non-motor symptoms. The relationship between COMT gene polymorphisms and PD is complex and not fully elucidated. Some studies have reported associations between certain COMT gene variants and PD risk, while others have not found significant associations. This study investigates how COMT gene variations impact cortical thickness changes in PD patients over time, aiming to link genetic factors, especially COMT gene variations, with PD progression. This study analyzed data from 44 PD patients with complete 4-year imaging follow-up from the Parkinson Progression Marker Initiative (PPMI) database. Magnetic resonance imaging (MRI) scans were acquired using consistent methods across 9 different MRI scanners. COMT single-nucleotide polymorphisms (SNPs) were assessed based on whole genome sequencing data. Longitudinal image analysis was conducted using FreeSurfer's processing pipeline. Linear mixed-effect models were employed to examine the interaction effect of genetic variations and time on cortical thickness, while controlling for covariates and subject-specific variations. The rs165599 SNP stands out as a potential contributor to alterations in cortical thickness, showing a significant reduction in overall mean cortical thickness in both hemispheres in homozygotes (Left: P = 0.023, Right: P = 0.028). The supramarginal, precentral, and superior frontal regions demonstrated significant bilateral alterations linked to rs165599. Our findings suggest that the rs165599 variant leads to earlier manifestation of cortical thinning during the course of the disease. However, it does not result in more severe cortical thinning outcomes over time. There is a need for larger cohorts and control groups to validate these findings and consider genetic variant interactions and clinical features to elucidate the specific mechanisms underlying COMT-related neurodegenerative processes in PD.

## Introduction

Parkinson's disease (PD) is a progressive neurodegenerative disorder affecting over 3% of the population aged over 65 years, which has garnered attention for centuries^1,2^. Its underlying mechanism involves a reduction in dopaminergic neurons within the substantia nigra, alongside the presence of Lewy bodies, leading to a combination of motor symptoms like rigidity, tremor, and bradykinesia, as well as non-motor symptoms including anxiety and depression^3,4^. Motor symptoms are attributed to dopaminergic neuron loss in the striatum, with nonmotor symptoms suggesting broader neuronal decline^5,6^.

The disease's origins are linked to environmental changes, genetic predisposition, and exposure to toxins. While a cure remains elusive, ongoing research explores treatments such as neuronal replacement, α‐synuclein, and apomorphine^3,4,7^.

Catechol-O-methyltransferase (COMT; EC 2.1.1.6) is vital for metabolizing catecholamines, key neurotransmitters in mammals. It adjusts dopamine, norepinephrine, and catechol estrogen levels by methylating them, facilitating their degradation, and impacting their activity in the body^8^.

COMT inhibitors like entacapone, opicapone, and tolcapone play an important role in PD treatment, particularly in patients experiencing motor fluctuations due to prolonged levodopa therapy. By blocking the COMT enzyme, they enhance levodopa's effectiveness due to slowing its breakdown in the body^9^.

7784 single-nucleotide polymorphisms (SNPs) exist within the COMT gene, with some having an impact on PD risk. Multiple studies have provided evidence suggesting a connection between certain alleles associated with low activity of the COMT enzyme and the risk of developing PD. Previous investigations have found that individuals who carry specific homozygous COMT alleles, such as rs4680 and rs4633, have a reduced risk of developing PD^10^.

A prior investigation into imaging-genetic associations regarding the age at onset of PD and fractional anisotropy in diffusion imaging revealed that the majority of variants influencing these outcomes are associated with the COMT gene. This suggests a potential involvement of the COMT gene in influencing both diffusion imaging fractional anisotropy and the age at onset of PD^11^.

Another prior investigation into COMT SNPs and their haplotypes, focusing on age-related changes in cortical thickness and subcortical shapes in large healthy adult samples independent of PD status, revealed an age-related decline in cortical thickness in specific brain regions and shape compression in the basal ganglia, with variations dependent on COMT genotypes and haplotypes. This study demonstrates that COMT SNPs can influence brain structure even in contexts unrelated to PD^12^.

This study aims to investigate how variations in the COMT gene influence changes in cortical thickness over time in individuals with PD. As neurodegeneration might start years before clinical PD diagnosis based on motor symptoms, we aim to understand the specific impact of COMT gene variations on temporal cortical thickness changes in PD patients^13^.

This analysis seeks to establish a connection between genetic factors, particularly COMT gene variations, and PD progression. Focusing on cortical thickness as a marker of brain structure changes, our objective is to uncover genetic influences on the neurodegenerative process in PD. This research could enhance our understanding of underlying mechanisms, shedding light on genetic contributions to PD's pathophysiology.

## Methods

### Subject selection

The Parkinson Progression Marker Initiative (PPMI) is a global research endeavor with the aim of identifying biomarkers for PD progression, contributing to a deeper comprehension of the disease and facilitating the success of therapeutic trials^14^. Participants with PD were selected from the PPMI database, specifically those who underwent complete 4-year imaging follow-up and received MRI scans at Baseline, visit 4 (month 12), visit 6 (month 24), and visit 10 (month 48). Inclusion criteria comprised T1 images acquired via the SIEMENS TrioTim scanner, characterized by a thickness of 1.0 mm, 3D Acquisition Type, and a Field Strength of 3.0 Tesla. After applying these criteria, 47 subjects were initially identified, though 3 were excluded due to a lack of genetic assessment, ultimately resulting in the inclusion of 44 subjects in the study. Additional information regarding genotyping and MRI acquisition is available on the PPMI website (http://ppmi-info.org). The data utilized in this manuscript were extracted from the PPMI database and accessed on July 07, 2023.

### MRI images

After applying the specified inclusion criteria, our study comprised 44 PD patients with available genetic data and a set of 4 MRIs acquired using a consistent methodology, resulting in a total of 176 images. Upon closer examination of the image acquisition method within this selected group, we identified that these images had been captured by 9 distinct MRI scanners employing the MPRAGE sequence. The acquisition parameters for these images were as follows: a consistent repetition time of 2300 ms across all 176 images; an echo time of 2.98 ms in 161 MRIs, 2.52 ms in 8 MRIs, and 2.96 ms in 7 MRIs; a uniform inversion time of 900 ms for all images; a steady flip angle of 9° for all images; a matrix size of [240 × 256] in 144 images, [256 × 256] in 24 images, and [246 × 256] in 8 images; and consistent voxel dimensions of 1 × 1 × 1 mm3. The majority of images originated from three distinct centers: the Center of Brain Imaging at the University of Marburg contributed 60 images, Emory University provided 40 images, and Baylor College of Medicine supplied 28 images.

### COMT SNPs

#### Source and description

The allelic status of specific variants linked to Parkinson's disease in PPMI subjects was previously evaluated by Matloff et al. They examined these variants using whole genome sequencing data, identifying the alleles present in individuals who had undergone this sequencing. By extracting genotypes from aligned VCF files (hg38) through the utilization of BCFtools, they focused on particular variants. The data in VCF format was subsequently converted into a binary PLINK format, with the application of a GQ score threshold. On August 25, 2023, we accessed this valuable dataset from the PPMI database^15^. The PPMI dataset includes 11 SNP mutation variants for the COMT Gene: rs165599, rs4680, rs4818, rs2239393, rs4633, rs6269, rs165656, rs740603, rs5993883, rs174674, and rs737866.

#### Clustering and Co-inheritance

Linkage disequilibrium, a crucial aspect of COMT polymorphism, involves the non-random association of alleles at different loci, impacting the analysis and interpretation of study results^16^. For instance, in white populations, SNPs rs4680 and rs4633 exhibit complete linkage (D′ = 1.00), meaning they are consistently inherited together without recombination events. Conversely, SNPs rs4680 and rs4818, as well as rs4633 and rs4818, show partial linkage (D′ = 0.685), indicating correlated but imperfectly linked inheritance, allowing for recombination events between them^10^.

Identifying co-occurring SNPs is paramount due to the significant influence of co-inherited COMT polymorphisms on result interpretation. To understand potential genetic relationships and interpretive patterns, we'll use the following methods:

#### Correlation analysis

The data will be re-coded numerically, with homozygous for reference allele cases as zero, heterozygous cases as 1, and homozygous for alternate allele cases as 2. Spearman correlation will then be conducted to determine which SNPs should be grouped together using threshold of 1.0.

#### LD analysis

For Linkage Disequilibrium computation, we utilized the method devised by Rogers and Huff^17^, which adjusts for nonrandom mating and yields estimates comparable to established algorithms like the EM estimator. Calculations were conducted using the scikit-allel library for Python^18^, while visualization was achieved through SRplot, a freely available online platform for data visualization and graphing^19^. An R2 of 1.0 is considered as the criteria for grouping.

#### Hierarchical clustering

As SNP identification grows, the computational load for haplotyping and epistasis analysis surges. Hierarchical clustering aids in exploring SNP patterns within a gene, offering insights into their groupings^20^. To identify tightly packed clusters with minimal within-cluster variance a Ward linkage was used. Distances between datapoint was calculated using Euclidean distance. A distance equal to zero was the criteria for grouping. SciPy library for Python used for this analysis.

### Neuroimaging analysis

Longitudinal image analysis has emerged as a pivotal approach for investigating typical aging and neurodegenerative conditions, providing valuable understandings of disease advancement and treatment strategies. To ensure impartial assessment across numerous time points, we employed FreeSurfer's longitudinal processing pipeline. This involved creating an unbiased within-subject template through iterative alignment with a median image using robust registration. This method not only minimizes variability by aligning all time points within a standardized voxel space, but also offers a reliable foundation for initializing subsequent segmentation processes^21,22^.

**Figure 1:**
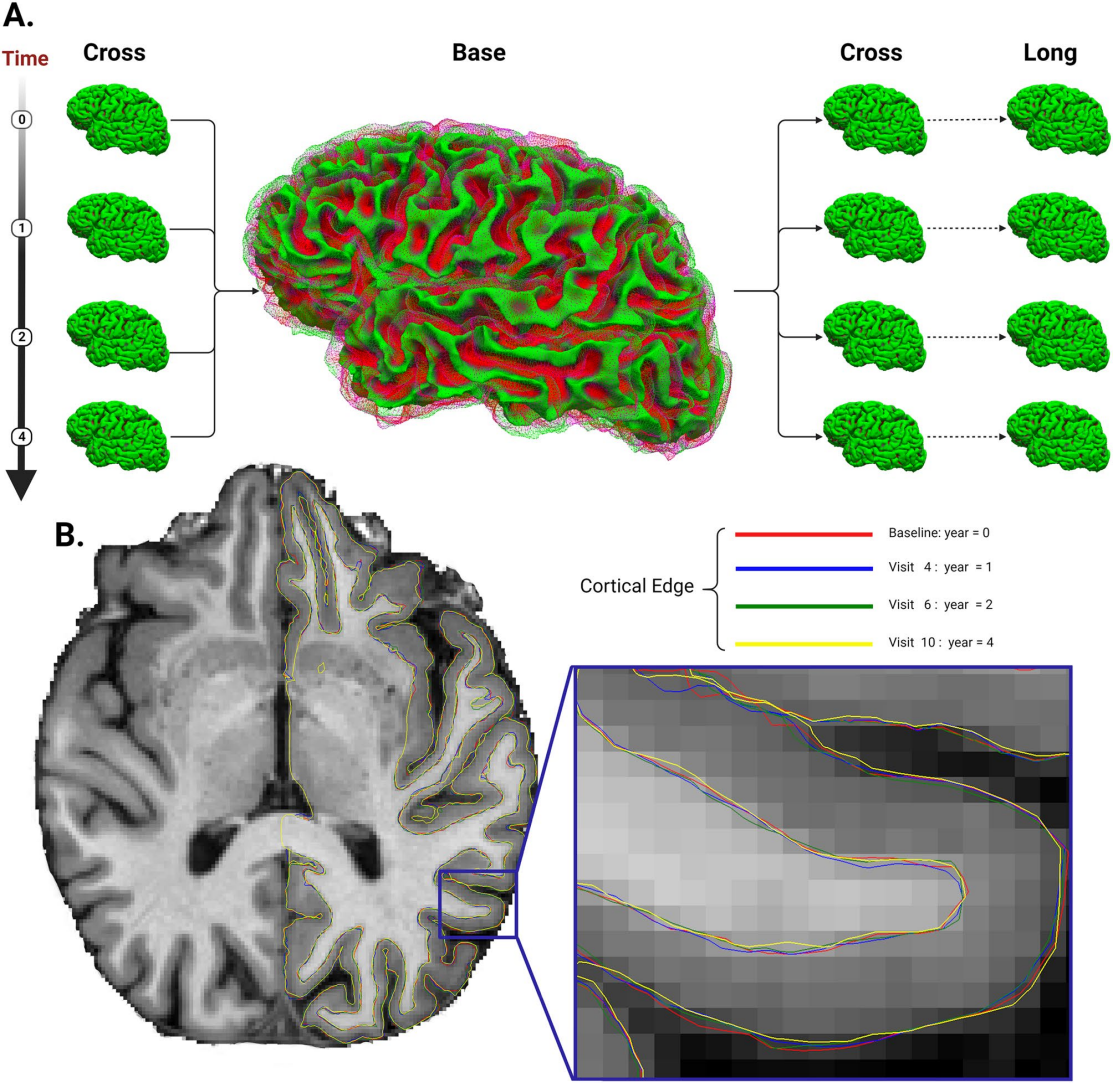
Analysis Workflow (**A**): The acquired MRI images were reconstructed to generate Cross images. Subsequently, four Cross images from each subject were aggregated to create an average anatomy template known as Base. The Base template, along with the longitudinally processed Long images at each time point, was used to create the final longitudinal images.; (**B**): The reconstructed Long surfaces are overlaid onto their respective Base templates.

In processing longitudinal MRI images, the Cross-Sectional (CROSS) step involves independent processing of all time points per subject, performing individual image segmentation and surface reconstruction while retaining data for later. The Subject Template (BASE) stage creates an average anatomy template using unbiased median images from all time points, including full segmentation and surface reconstruction. In Longitudinal (LONG) Processing, each time point is longitudinally processed, utilizing both the subject template [BASE] and individual runs [CROSS] to initialize algorithms, leading to heightened sensitivity and repeatability compared to standalone cross-sectional runs^21^. This workflow is summarized in Fig. 1.

### Statistical analysis

After generating longitudinal images, regional statistics for cortical thickness were derived using the DKT atlas Region of Interest (ROI) approach. Given the multifactorial nature of brain cortical thickness changes and the need to control for confounding variables while assessing the impact of Time and Genetic variation on brain cortical thickness, a linear mixed effect model (LMM) approach was adopted.

In our LMM analysis, we examined the cortical thickness of brain segments obtained from longitudinal MRI scans as the dependent variable. The central focus was on the interaction effect between the genetic GROUP designation and TIME. The GROUP variable was categorized into three states: Absent mutation, heterozygote mutation, and homozygote mutation. To comprehensively account for potential confounders, the model incorporated covariates including Sex, Weight, within-subject effects, and between-subject effects.

To mitigate the impact of inter-subject brain size variations, we introduced the estimated total intracranial volume (eTIV) as a predictor in the model. Our underlying hypothesis was that brain volume might influence cortical thickness, with this effect likely varying across different brain regions. Given the longitudinal nature of the study with four repeated measures per subject, it was crucial to address subject-specific variations. Consequently, a mixed-effects structure was implemented, assigning a random effect to each subject to capture their unique characteristics:

$${\text{Cortical}} - {\text{Thickness }}\sim {\text{ Sex }} + {\text{ Weight }} + {\text{ TIME}}*{\text{GROUP }} + \, \left( {{1 }|{\text{ Subject}}} \right) \, + {\text{ eTIV}}$$

After assessing the effect of time and genetic variations on cortical thickness in all segments, the results were reported when there was a meaningful overall effect of genetic variations on cortical thickness or a meaningful effect of genetic variations on cortical thickness alteration rates.

To assess the goodness of fit or the explanatory power of the LMM model, we employed "Marginal R-squared" and "Conditional R-squared." Marginal R^2^ gauges the fraction of variance accounted for by the fixed effects, akin to the traditional R^2^ in linear regression, while it disregards random effects. Conversely, Conditional R^2^ takes into consideration both the fixed and random effects, providing a more comprehensive evaluation by encompassing all sources of variability. It frequently surpasses Marginal R^2^ due to the inclusion of the explanatory contribution of random effects^23,24^.

The statistical analysis was performed in R using the lme4^25^ and Plotly packages, allowing for the implementation of the LMM and visualization of the results. Estimated marginal means are reported as EMM ± Standard error of the mean (SEM).

#### Controlling false discoveries

In this study, a statistical model is developed for every SNP across 70 regions, totaling up to 770 models (11 SNPs and 70 regions). Each model explores at least 5 desirable hypotheses. This approach leads to a significant number of simultaneous statistical tests, which raises the risk of Type I errors or false positives, presenting the Multiple Comparisons Problem. To counteract this issue,

To combat the challenge of multiple comparisons, False Discovery Rate (FDR) correction was implemented, specifically the approach pioneered by Yoav Benjamini and Yosef Hochberg (BH) was utilized. This method offers a more flexible and potent means of hypothesis testing, managing Type I error inflation without overly conservative adjustments by controlling the FDR. Implementation of the Benjamini–Hochberg procedure was carried out using the statistical software R^26,27^.

## Results

### Clustering

**Figure 2:**
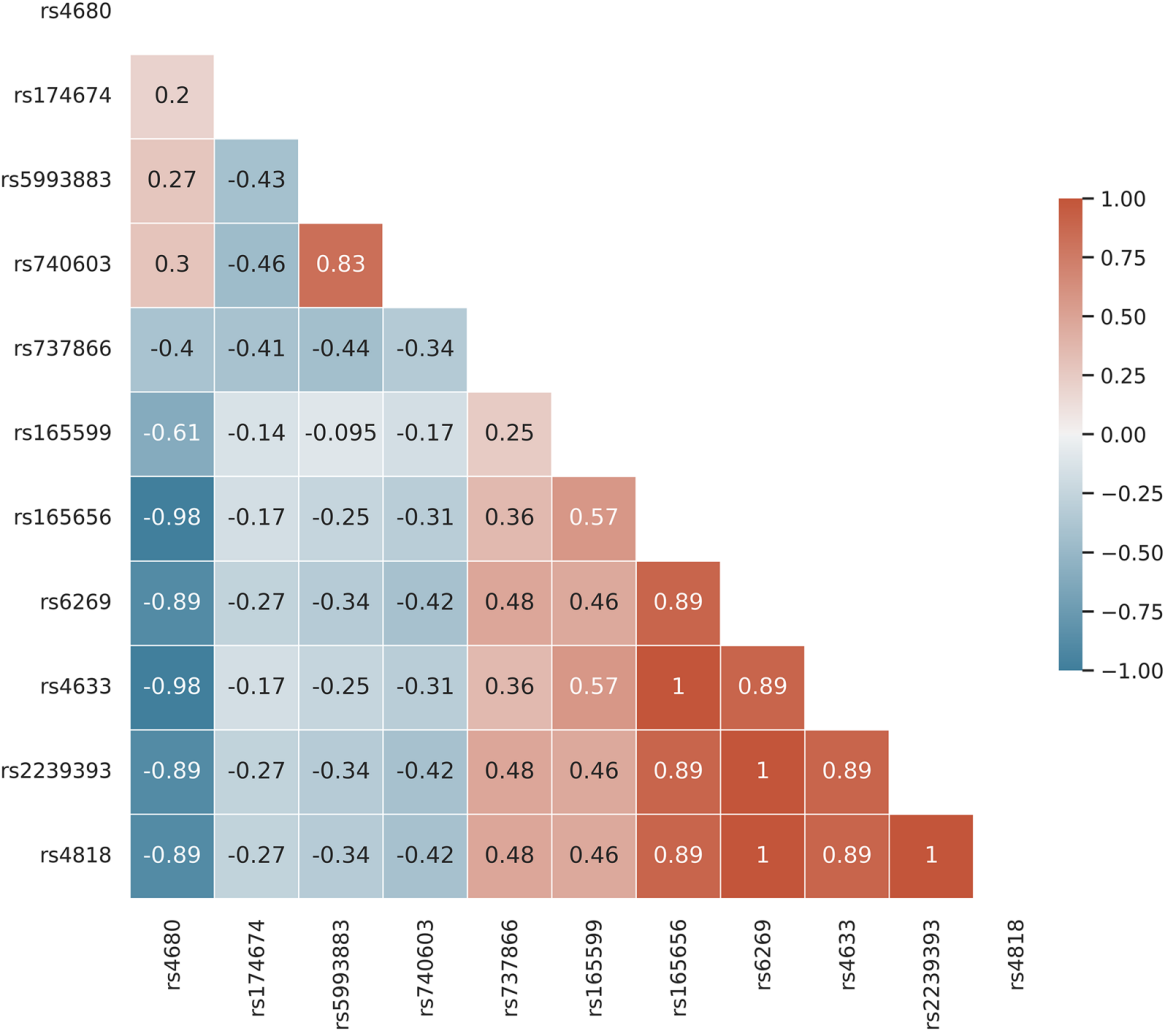
COMT Variations Co-inheritance. Spearman correlation analysis on the number of inherited COMT alleles.

Given the notable tendency of COMT polymorphisms to be co-inherited, this inherent phenomenon bears substantial influence on result interpretation. Consequently, our foremost endeavor involves identifying the co-occurring SNPs. To substantiate the existence of alleles contingent upon the presence of other alleles, we conducted a Spearman correlation analysis on the number of inherited alleles within each subject (see Fig. 2).

As depicted in Fig. 2, it is evident that rs2239393, rs6269, and rs4818 exhibit a perfect correlation coefficient of 1, implying their consistent co-occurrence, effectively rendering them identical variables. Similarly, rs4633 and rs165656 also demonstrated a correlation coefficient of 1, indicating their indistinguishable nature. On the contrary, rs4633 and rs165656 exhibited an extraordinarily strong negative correlation coefficient of -0.98 with rs4680, implying that their homozygous states never occurred concurrently. However, it's essential to note that while this data and analysis reveal that these particular alleles rarely co-occurred, we cannot definitively conclude that all alleles never existed together. Therefore, we conducted a separate analysis for rs4680.

**Figure 3:**
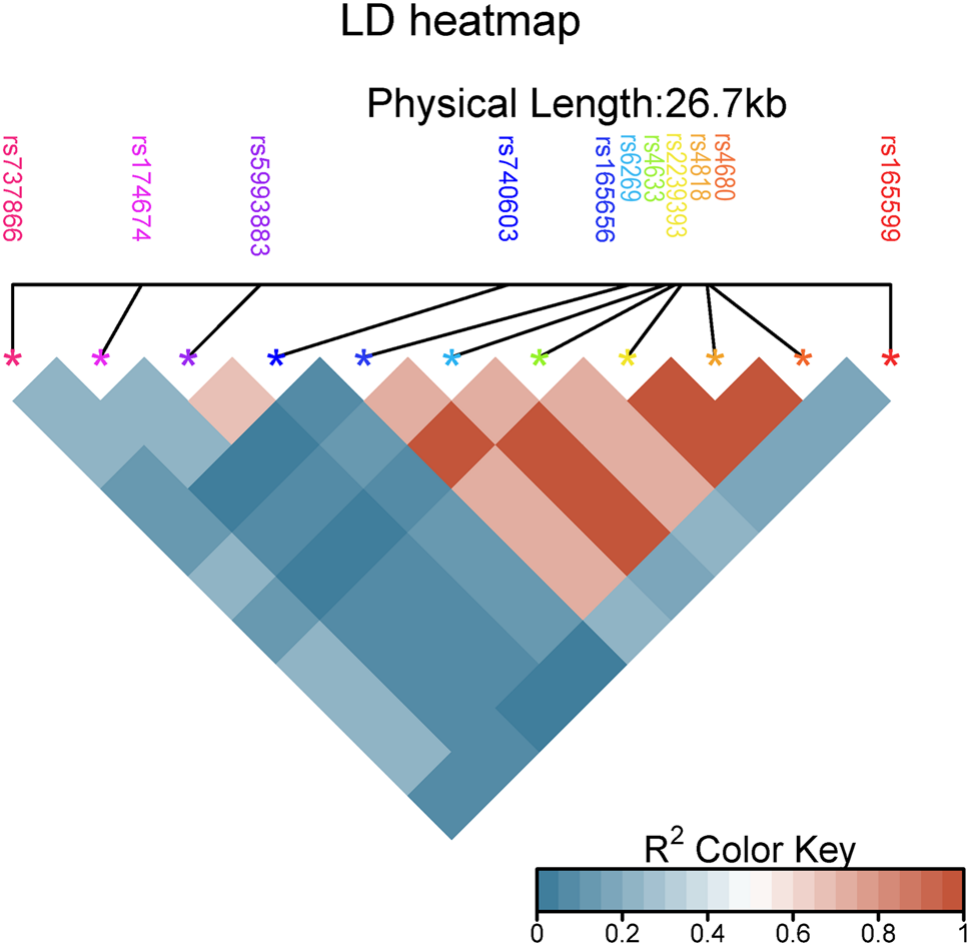
Linkage Disequilibrium Heat Map. LD, measured by r2, is shown in a triangular heatmap. Colors denote r2 values as per the key, with SNP sites marked by colored stars on gene model.

The results of the linkage disequilibrium analysis are depicted in Fig. 3. As illustrated, the SNPs rs2239393, rs6269, and rs4818 are in close proximity, with a physical distance of 476 base pairs. Their high linkage disequilibrium coefficient (R2 = 1.0) suggests that they can be considered as a single entity. Similarly, rs4633 and rs165656 exhibit proximity, with a distance of 1372 base pairs and an R2 of 1.0.

**Figure 4:**
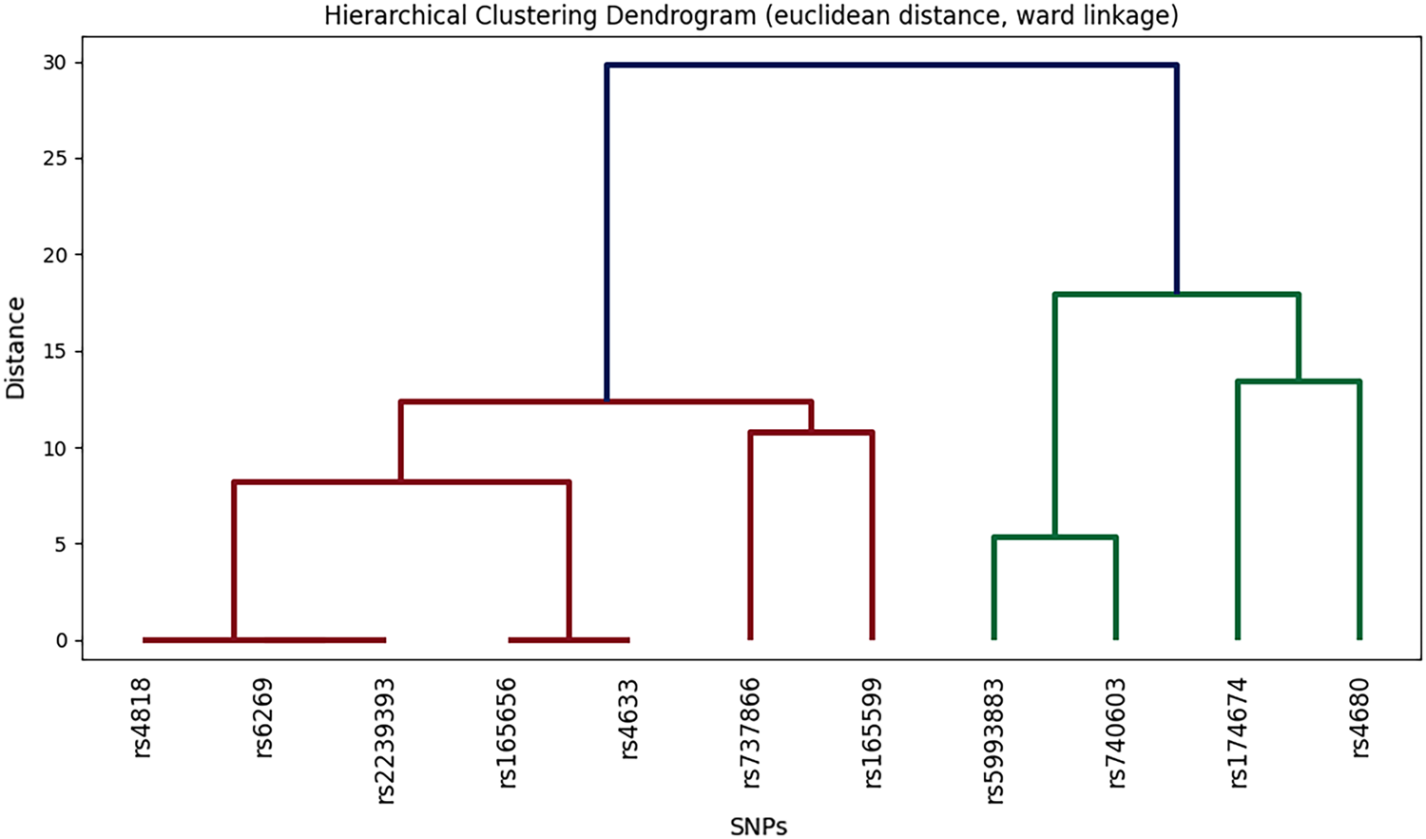
Hierarchical clustering diagram. This diagram illustrates the results of hierarchical clustering analysis conducted on SNP patterns within the COMT gene. The Ward linkage method and Euclidean distance were employed for clustering. A grouping criterion of zero distance was used to merge identical SNPs into single groups.

The hierarchical clustering results are depicted in Fig. 4. As observed, rs2239393, rs6269, and rs4818 exhibit zero Euclidean distance, indicating their close proximity. Similarly, rs4633 and rs165656 also demonstrate zero Euclidean distance, suggesting their suitability for being grouped together as one cluster.

### rs165599

Among 44 subjects, 20 (45.5%) had no rs165599 mutations, 20 (45.5%) were heterozygotes with one mutation, and 4 (9.1%) were homozygotes with two mutations. For those without rs165599, the COMT gene averaged 6.65 concurrent SNPs. Heterozygotes showed 8.70, and homozygotes had 9.75 other simultaneous COMT SNPs on average. The summary of results pertaining to the influence of the rs165599 mutation on cortical thickness is presented in Table 1 for symmetric changes occurring on both hemispheres and in Table 2 for asymmetric changes occurring exclusively on one side.

**Table 1:** Symmetric alterations in cortical thickness associated with rs165599.

Cortex	Overall Effect of rs165599				Effect of rs165599 on Temporal alteration Rate						R2: Marginal/ Conditional
	HETERO		HOMO		Time		Time *HETERO		Time *HOMO		
	Coeff	P	Coeff	P	Coeff	P	Coeff	P	Coeff	P	
L-Mean	10.6	.911	− 119	**.023**	− 4.12	.062	− 4.97	.344	.62	.951	.227/.901
R-Mean	8.98	.911	− 108	**.028**	-5.61	**.031**	− 2.63	.694	2.60	.797	.179/.875
L-Postcentral	32.7	.875	− 150	**.023**	− 1.93	.653	− 10.5	.233	− 1.84	.930	.318/.853
R-Postcentral	46.1	.875	− 169	**.002**	− 9.26	**.006**	− 4.56	.596	6.84	.628	.342/.873
L-Supramarginal	22.9	.911	− 146	**.023**	− 3.01	.310	-10.7	.070	− 3.32	.797	.273/.899
R-Supramarginal	6.0	.936	− 136	**.040**	− 9.4	**.010**	− 4.46	.635	5.04	.757	.164/.861
L-Superior frontal	24.1	.911	− 188	**.002**	− 1.06	.747	− 2.81	.761	9.39	.478	.364/.907
R-Superior frontal	36.4	.875	− 146	**.023**	− 1.18	.747	.61	.970	8.60	.619	.329/.885

The bold p-values denote statistically significant results at the 0.05 significance level.

HETERO: Heterozygote or subjects with only one allele with rs165599 mutation; HOMO: Homozygote or subjects with two rs165599; L: Left; R: Right; Coeff: Coefficient; P: P Value.

**Table 2:** Asymmetric alterations in cortical thickness associated with rs165599.

Cortex	Overall Effect of rs165599				Effect of rs165599 on Temporal alteration Rate						R2: Marginal/ Conditional
	HETERO		HOMO		Time		Time *HETERO		Time *HOMO		
	Coeff	P	Coeff	P	Coeff	P	Coeff	P	Coeff	P	
L- Middle temporal	− 24.7	.911	− 200	**.028**	− 10.9	**.006**	0.77	.970	− 2.54	.912	.201/.902
L-Pars-orbitalis	11.8	.911	− 89.4	.373	5.93	.220	− 8.48	.481	− 29.6	**.035**	.166/.887
R-Temporal pole	− 52.2	.911	-310	**.028**	− .71	.921	− 20.6	.344	− 18.2	.628	.232/.815
L-Superior parietal	41.1	.875	− 142	**.032**	− 1.49	.730	− 9.53	.280	0.57	.960	.246/.868
L-Rostral middle frontal	− 16.0	.911	− 106	.089	1.80	.653	− 1.85	.856	23.9	**.013**	.087/.828
L-Precuneus	57.6	.875	− 148	**.028**	− 7.41	**.050**	− 8.69	.344	8.76	.628	.254/.856

The bold p-values denote statistically significant results at the 0.05 significance level.

HETERO: Heterozygote or subjects with only one allele with rs165599 mutation; HOMO: Homozygote or subjects with two rs165599; L: Left; R: Right; Coeff: Coefficient; P: P Value.

#### Mean cortical thickness

**Figure 5:**
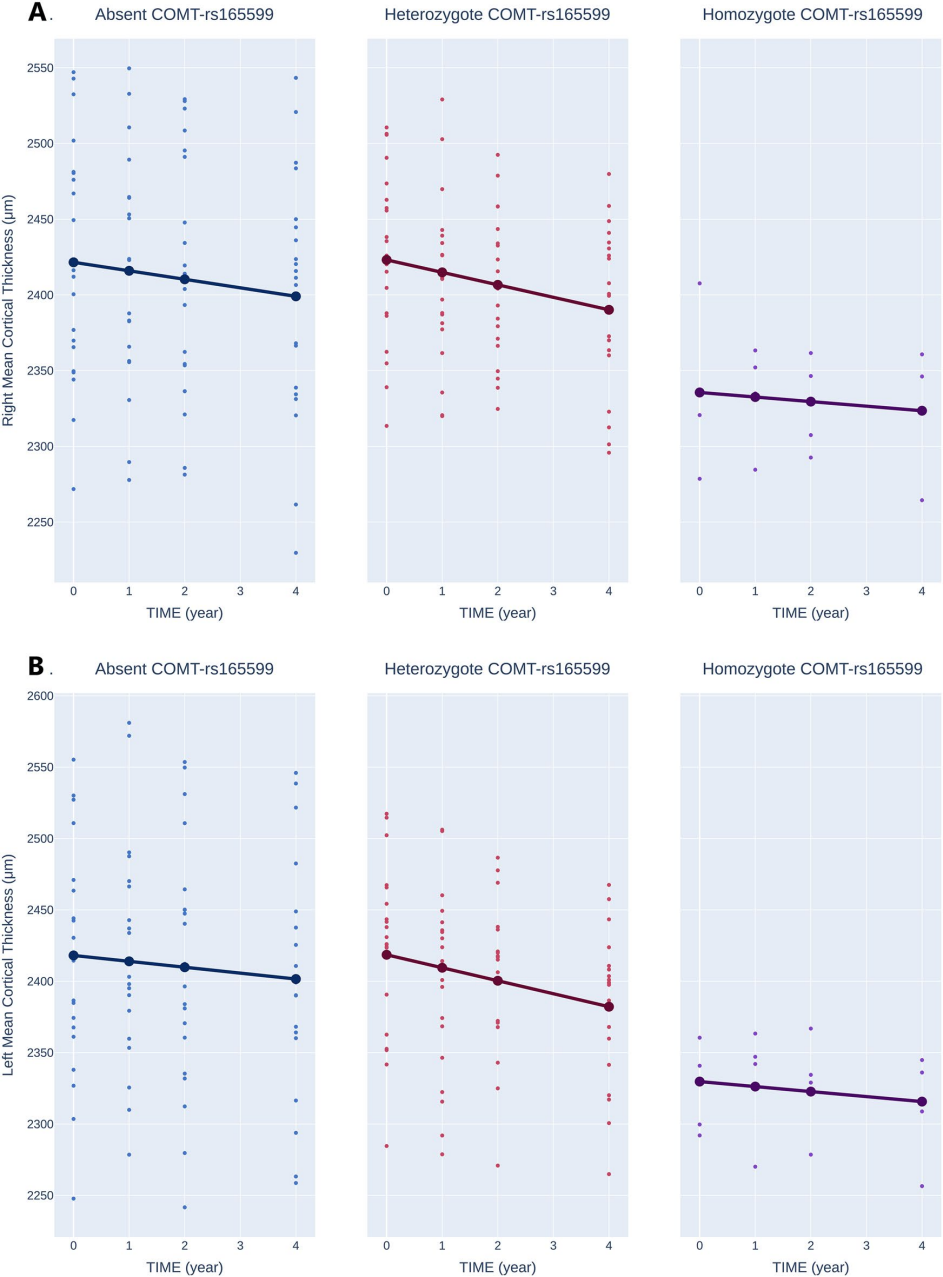
Effects of rs165599 on Mean Cortical Thickness. (**A**): right hemisphere, (**B**): left hemisphere.

The average cortical thickness of the left hemisphere was measured at 2399.18 ± 5.43 µm. After accounting for covariates and FDR correction, it was observed that time had no significant impact on the average cortical thickness of the left hemisphere (*P* = 0.062). Among heterozygotes, the mean cortical thickness of the left hemisphere measured 2422 ± 16.1 µm, showing no significant difference from other cases (*P* = 0.911). However, among Homozygotes, the mean cortical thickness of the left hemisphere exhibited a significant decrease of 119.02 ± 37.77 µm (*P* = 0.023). There were no significant differences in the annual changes in thickness in heterozygotes (*P* = 0.344) or Homozygotes (*P* = 0.951). See Fig. 5B.

In the right hemisphere, the mean cortical thickness was also 2402.91 ± 5.20 µm. After adjusting for covariates and FDR correction, time was found to have a significant impact, resulting in a yearly reduction of 5.62 ± 1.93 µm (*P* = 0.031) in cortical thickness. In heterozygotes, the cortical thickness was 8.98 ± 20.8 µm higher, although this difference was not statistically significant (*P* = 0.911). In contrast, Homozygotes showed a significant reduction of 107.89 ± 37.10 µm in cortical thickness (*P* = 0.028). As in the left hemisphere, there were no significant differences in the annual changes in thickness in heterozygotes (*P* = 0.694) or Homozygotes (*P* = 0.797). See Fig. 5A.

#### Symmetric changes

##### Postcentral cortex

Homozygotes exhibited notably reduced cortical thickness, with a significant difference of 150.59 ± 45.0 µm lower in the left postcentral cortex (P = 0.023) and -169.87 ± 43.1 µm lower in the right postcentral cortex (P = 0.002). In contrast, heterozygotes did not display any significant overall differences in cortical thickness.

##### Supramarginal cortex

Individuals homozygous for the rs165599 mutation showed a significant and symmetrical reduction in cortical thickness, with a decrease of 146.63 ± 46.9 µm in the left Supramarginal Cortex (*P* = 0.023) and 135.97 ± 49.4 µm in the right Supramarginal Cortex (*P* = 0.040). In contrast, heterozygotes did not exhibit any significant alterations in cortical thickness compared to the mean Supramarginal thickness.

##### Superior frontal cortex

There was no significant temporal effect observed in either hemisphere within this region. Subjects carrying the rs165599 variant, similar to others, did not display any discernible temporal trends in cortical thickness alterations. However, individuals with two mutated rs165599 genes exhibited a significantly lower mean thickness in the Superior frontal cortex. In the left hemisphere, homozygotes showed a substantial thinning of the cortex, with a thickness reduction of 188.39 ± 46.4 µm (*P* = 0.002), and in the right hemisphere, homozygotes exhibited a significant cortical thinning of 145.6 ± 46.9 µm (*P* = 0.023). In contrast, individuals with rs165599 heterozygosity did not exhibit any differences in mean cortical thickness compared to non-mutation carriers.

#### Asymmetric changes

##### Left Middle temporal Cortex

A significant main temporal effect was observed, showing a reduction of 10.9 ± 3.03 µm/year (*P* = 0.006). However, carrying the rs165599 mutation did not influence this trend. Having two mutated rs165599 alleles was associated with a substantial reduction of 200.47 ± 65.6 µm in the mean thickness of the Left Middle Temporal Cortex (*P* = 0.028). On the other hand, being a heterozygote for this variation had no significant effect (*P* = 0.911).

##### Left pars-orbitalis cortex

A significant main temporal effect was not detected. Nonetheless, homozygosity was associated with a notable increase in the temporal reduction rate of 29.6 ± 9.1 µm/year (*P* = 0.035). In contrast, carrying the rs165599 allele did not exhibit any significant impact on the overall mean thickness of the left Pars-Orbitalis cortex.

##### Right temporal pole cortex

A significant main temporal effect was not detected. Homozygosity was associated with a substantial reduction of 310 ± 106.9 µm in Right Temporal Pole thickness compared to the overall mean (*P* = 0.028).

##### Left superior parietal cortex

Homozygosity for the rs165599 mutation was associated with a significant reduction in Left Superior Parietal thickness, showing a decrease of 141.6 ± 49.7 µm (*P* = 0.032). On the other hand, individuals with heterozygous rs165599 mutations did not exhibit any significant differences in cortical thickness.

##### Left rostral middle frontal cortex

There were no significant main temporal effects observed in either hemisphere within this region. In the left hemisphere, homozygotes exhibited a notable increase in Rostral middle frontal cortex temporal alteration rates, with a rate increment of 23.93 ± 6.58 µm/year (*P* = 0.013).

##### Left precuneus cortex

Homozygosity for the rs165599 mutation was linked to a significant reduction in Left Precuneus thickness, with a decrease of 147.9 ± 50.8 µm in the left hemisphere (*P* = 0.028). Overall, significant temporal effects were observed in Precuneus thickness alterations over time in left Precuneus (-7.41 ± 3.0 µm/year, *P* = 0.050) but carrying rs165599 mutation had no effect on this trend.

### rs2239393, rs6269, and rs4818 (Group2)

These mutations occurred in a consistent manner, prompting their joint evaluation. Among the 44 subjects, 18 (40.9%) did not possess mutations for rs2239393, rs6269, and rs4818, while 21 (47.7%) were heterozygotes, each carrying one mutation for rs2239393, rs6269, and rs4818 SNPs. Additionally, 5 subjects (11.4%) were homozygotes, carrying two mutations for each of these SNPs.

For those without these SNPs, the COMT gene had an average of 5.67 concurrent SNPs. Heterozygotes exhibited an average of 8.81 concurrent SNPs, taking into account identical alleles, and 6.81 without considering them. In contrast, homozygotes had an average of 11.2 other concurrent COMT SNPs, considering identical alleles, and 7.2 without considering them. To facilitate the discussion of these SNPs, we will collectively refer to rs2239393, rs6269, and rs4818 as "Group2 SNPs."

#### Mean cortical thickness

The estimated marginal mean (EMM) cortical thickness of the left hemisphere was 2405 ± 17.4 µm in heterozygotes and 2412 ± 32.6 µm in homozygotes. Consequently, carrying Group2 genes did not result in a significant difference in the overall mean thickness of the left hemisphere (*P* = [0.946, 0.996]).

Similarly, in the right hemisphere, the EMM cortical thickness was 2405 ± 16.6 µm in heterozygotes and 2422 ± 31.0 µm in homozygotes. Once again, carrying Group2 genes did not yield a significant difference in the overall mean thickness of the right hemisphere (*P* = [0.946, 0.973]).

While we did observe a significant primary temporal effect, indicating a reduction of 7.0 ± 1.94 µm/year in the left hemisphere (*P* = 0.001) and 7.97 ± 2.06 µm/year in the right hemisphere (*P* < 0.001), it's important to note that carrying Group2 genes did not influence this trend.

#### Regional changes

After correcting for False discovery rates using Benjamini–Hochberg procedure, no regional changes were associated with carrying Group2 SNPs and overall thickness at baseline or thickness loss rate over time. See supplementary data section for Complete results review and before and after FDR correction *P* values.

### rs4633 and rs165656 (Group3)

Among the 44 individuals studied, 16 (36.4%) lacked mutations in both rs4633 and rs165656, while 18 (40.9%) were heterozygous, carrying one mutation for each of these SNPs. In addition, 10 individuals (22.7%) were homozygous, carrying two mutations for both rs4633 and rs165656 SNPs.

For those without these specific SNPs, the COMT gene exhibited an average of 5.37 concurrent SNPs. Heterozygotes, when accounting for identical alleles, showed an average of 8.22 concurrent SNPs, which decreased to 7.22 when not considering identical alleles. In contrast, homozygotes had an average of 10.2 other concurrent COMT SNPs, with identical alleles considered, and 8.2 without considering identical alleles. To simplify the discussion of these SNPs, we will collectively refer to rs4633 and rs165656 as "Group3 SNPs."

#### Mean cortical thickness

A significant reduction trend was observed in both hemispheres. However, carrying the Group3 variation did not influence this trend and did not have any effect on the overall mean cortical thickness.

#### Regional changes

After adjusting for False Discovery Rates using the Benjamini–Hochberg procedure, no regional changes were found to be linked with carrying Group3 SNPs in terms of either baseline overall thickness or the rate of thickness loss over time. See Complete results in supplementary materials.

### rs174674

Among the 44 subjects, 18 (40.9%) lacked rs174674 mutations and showed an average of 9.94 concurrent COMT SNPs. Additionally, 20 (45.5%) were heterozygotes, carrying one mutation, and had an average of 6.5 concurrent COMT SNPs, while 6 (13.6%) were homozygotes with two mutations, demonstrating an average of 5.50 concurrent COMT SNPs.

#### Mean cortical thickness

Consistent with previous findings, time had a negative impact on both hemispheres (*P* < 0.001), leading to a reduction in thickness over time. The presence of the rs740603 mutation did not show any significant alterations in the temporal effect in either hemisphere. Furthermore, individuals carrying the rs740603 mutation did not display a significant difference in mean cortical thickness compared to those without the mutation.

#### Regional changes

In the left Posterior Cingulate cortex having one rs174674 mutation was associated with a decrease in the overall mean thickness (− 120 ± 29.9 µm, *P* = 0.003). Other regions showed no association with rs174674 mutation.

### rs740603, rs5993883, rs737866 and rs4680

All these SNPs were analyzed independently. A significant decreasing trend in mean cortical thickness over time in both hemispheres were observed same as before; However, carrying these variations did not impact this trend nor affect the overall mean cortical thickness.

Upon adjusting for False Discovery Rates, no significant associations were detected between carrying these SNPs and changes in baseline cortical thickness or alterations in its trend over time. Complete results can be found in the supplementary Table S1.

## Discussion

We have identified an association between the COMT polymorphism and a range of changes in both the initial thickness of the cortex and its alteration over time. Nevertheless, it's important to note that COMT SNPs do not inherit independently, and they exhibit strong linkage disequilibrium. This underscores the need for more comprehensive research to precisely identify the specific SNP responsible for these observed alterations.

The initial suspect for the alterations associated with the COMT polymorphism was rs165599, primarily because it was the sole SNP where its homozygous inheritance demonstrated a significant symmetric reduction in the whole cortical thickness in both the left (*P* = 0.023) and right (*P *= 0.028) hemispheres.

Heterozygosity for rs174674 mutation was linked to reduced mean thickness in the left Posterior Cingulate cortex. However, if individuals with one copy of the mutation experienced a notable change in cortical thickness while those with two copies did not, it would be unjustifiable to attribute rs174674 as the cause of the observed alterations. Additionally, rs174674 showed no associations with overall mean cortical thickness alterations.

Mutations in rs2239393, rs6269, and rs4818 (Group 2) did not influence the overall cortical thickness or the primary temporal reduction trend, mirroring the similar findings observed for mutations in rs4633 and rs165656 (Group 3). Similarly, mutations in rs740603, rs5993883, rs737866, and rs4680 did not affect the trends in cortical thickness.

Homozygosity for rs165599 was linked to a significant symmetric reduction in cortical thickness observed in the Postcentral, Supramarginal, and Superior frontal regions, as well as bilateral decreases in the overall mean cortical thickness.

The homozygous rs165599 mutation was linked to an asymmetrical reduction in cortical thickness observed in the Left Middle Temporal, Left Precuneus, Left Superior Parietal, and Right Temporal Pole regions. Homozygous rs165599 was also associated with accelerated cortical loss in the Left Pars-Orbitalis but showed a protective effect against cortical loss in the Left Rostral Middle Frontal region.

To the best of our knowledge, this research marks the first investigation into the influence of COMT variations on cortical thickness trends in PD patients.

A study of 425 patients, including incident and prevalent cohorts, analyzed the relationship between COMT genotype and cognitive function (TOL scores). It confirmed a negative impact of increasing Met alleles (rs4680) on TOL scores. The study divided patients into early and later disease groups based on median disease duration and found a significant decline in TOL scores with more Met alleles in early disease stages, but not in later stages^28^. Our study did not replicate this connection for rs4680 and cortical thickness. Instead, we observed a significant decrease in Mean cortical thickness in patients with 2 rs165599 mutations. Furthermore, patients with fewer rs165599 mutations exhibited higher yearly reduction rates, though this disparity in reduction rate was not statistically significant after correction for false discovery rate.

Both our present study and the previously mentioned one suggest that the impact of COMT mutations on cortical thickness and cognition is most pronounced during the early stages of the disease, diminishing in significance over time^28^.

A study investigating chemotherapy-induced cognitive impairment in breast cancer patients identified a potential association between the COMT (rs165599) polymorphism and cognitive decline in breast cancer survivors. This suggests that rs165599 may impact cognition, cortex, and limbic system even in contexts other than Parkinson's disease^29^.

In a study investigating the impact of COMT SNPs on age-related changes in brain morphology among 214 healthy Singaporean Chinese volunteers, it was found that the rs737865-val158met-rs165599 haplotype played a significant role in modulating the association between age and cortical thickness. This modulation was observed in several regions, including the superior frontal cortex (SFC), orbitofrontal cortex (OFC), dorsolateral prefrontal cortex (dlPFC), middle temporal gyrus (MTG), posterior cingulate cortex (PCC), lingual cortex, and various other cortical areas. Specifically, the G-met-A haplotype was associated with altering the negative relationship between age and cortical thickness in the left SFC, OFC, dlPFC, MTG, PCC, and lingual cortex. Meanwhile, the G-val-A haplotype influenced the negative relationship between age and cortical thickness in the left inferior temporal cortex^12^.

These findings align with our own observations regarding the impact of rs165599 on cortical thickness. This underscores the importance of conducting further investigations to compare the influence of rs165599 between Parkinson's disease patients and a control group, particularly concerning clinical cognitive outcomes.

Among symmetrically affected regions by rs165599, the superior frontal cortex, particularly its lateral and posterior portions, plays a critical role in working memory (WM) processing, especially in tasks requiring high executive demands. Lesions in the lateral and posterior superior frontal cortex, notably area 8, result in significant deficits in WM, highlighting its hybrid nature with a preference for executive demands and relative spatial orientation^30^.

The supramarginal cortex plays a role in both verbal working memory and phonological processing, particularly in storing phonological representations rather than directly processing complexity. Specifically, the anterior supramarginal cortex is involved in domain-general verbal working memory processes, indicating its role in storing phonological information^31^. it is also found to be associated with feelings of empathy and overcoming emotional egocentric biases^32^.

The postcentral cortex contains the primary somatosensory cortex, crucial for proprioception and processing diverse somatic sensations like touch, pressure, temperature, and pain^33^.

This study faces limitations including a small sample size of 44 patients from the PPMI database. A larger sample size would enhance the statistical power and reliability of the results.

A study utilizing resting-state fMRI data from 120 women revealed that individuals who were homozygous for either rs4680, rs165599, or both SNPs showed a link between neuroticism and lower efficiency coefficients in visual and somatosensory-motor subnetworks. This means that the brain regions responsible for processing visual and somatosensory-motor information may not be effectively communicating or functioning optimally in individuals with these genetic traits^34^.

This aligns with the results of this study as we also found that rs165599 affects Post central cortex which is the key area for somatosensory-motor processing.

A meta-analysis of 20 case–control studies examining the association between the COMT gene rs165599 SNP and schizophrenia revealed no significant correlation between rs165599 SNP and schizophrenia across diverse populations^35^.

Another study investigating the association between the rs165599 polymorphism in the COMT gene's 3' untranslated region and methamphetamine addiction in a Taiwanese population, found no direct genotype differences but revealing a significant haplotype effect with rs4680, suggesting a potential regulatory role of rs165599 possibly through microRNA binding, and identifying evidence for antisense interference from a neighboring gene in the COMT 3'UTR^36^.

A study of COMT polymorphisms in modulating working memory in individuals with schizophrenia found that The COMT SNP rs165599, in combination with rs4680, showed a significant association with schizophrenia susceptibility, with the G-A haplotype being a risk factor. Additionally, rs165599 had opposite effects on cognitive performance in patients and controls, suggesting its role in modulating cognitive function in both schizophrenia and healthy subjects^37^.

This is consistent with our study's findings, as we observed that rs165599 influences brain regions associated with working memory.

This study highlights a significant influence of rs165599 on cortical thickness in Parkinson's disease patients. However, similar effects were also noted in healthy individuals, and the absence of change in the annual thinning rate complicates the definitive conclusion regarding rs165599's actual impact. Future research should prioritize comparing the effects of rs165599 between PD patients and healthy controls to better elucidate its role. The absence of a control group in this study presents challenges in clearly delineating the effect of COMT polymorphisms on PD patients.

Please note that although this study identifies associations between COMT gene variations and cortical thickness changes, it cannot establish causality. Other unmeasured factors could be driving these observed associations.

This study primarily focuses on genetic factors and cortical thickness changes, without a comprehensive assessment of the clinical implications or correlations with PD symptoms and progression. This could be the subject of future studies.

To mitigate the risk of publication bias arising from reporting only significant results, and to ensure a comprehensive representation of the data, we have included an Excel sheet in the supplementary data section, containing results for all regions and mutations.

## Conclusion

This longitudinal study, which explores the genetic influence of COMT gene variations on four-year temporal changes in cortical thickness in Parkinson's disease, has provided valuable insights into the complex interplay between genetics and neurodegenerative processes. While this research has shed light on certain associations, it is essential to acknowledge the limitations and nuances within our findings.

This study suggests that individuals with more rs165599 muted COMT genes may experience cortical thinning and cognitive loss earlier in disease course, but its severity does not escalate over time compared to others, and at later stages the difference became insignificant.

We have observed associations between specific COMT gene variations, particularly the rs165599 SNP, and alterations in cortical thickness in various brain regions. Notably, the supramarginal cortex, precentral cortex, and superior frontal cortex exhibited significant bilateral changes linked to COMT polymorphism. These findings suggest that genetic factors may contribute to the structural changes seen in the brains of individuals with Parkinson's disease.

Our research serves as a preliminary exploration into the impact of COMT gene variations on cortical thickness in PD patients. It underscores the need for more extensive investigations with larger and more diverse cohorts, considering potential interactions between genetic variants, and clinical features.

### Supplementary Information

Supplementary Information.

## References

[1] Braak H, Del Tredici K (2017). Neuropathological staging of brain pathology in sporadic Parkinson's disease: Separating the Wheat from the chaff. J. Parkinsons Dis..

[2] Raza C, Anjum R (2019). Parkinson's disease: Mechanisms, translational models and management strategies. Life Sci..

[3] Coon S, Stark A, Peterson E, Gloi A, Kortsha G, Pounds J (2006). Whole-body lifetime occupational lead exposure and risk of Parkinson's disease. Environ. Health Perspect..

[4] Maiti P, Manna J, Dunbar GL (2017). Current understanding of the molecular mechanisms in Parkinson's disease: Targets for potential treatments. Transl. Neurodegeneration..

[5] Zhou C, Huang Y, Przedborski S (2008). Oxidative stress in Parkinson's disease: a mechanism of pathogenic and therapeutic significance. Ann. N Y Acad. Sci..

[6] Ryman SG, Poston KL (2020). MRI biomarkers of motor and non-motor symptoms in Parkinson's disease. Parkinsonism Related Disorders..

[7] DeMaagd G, Philip A (2015). Parkinson's disease and its management: Part 1: Disease entity, risk factors, pathophysiology, clinical presentation, and diagnosis. P t..

[8] Chen J, Lipska BK, Halim N, Ma QD, Matsumoto M, Melhem S (2004). Functional analysis of genetic variation in catechol-O-methyltransferase (COMT): effects on mRNA, protein, and enzyme activity in postmortem human brain. Am. J. Hum. Genet..

[9] Fabbri M, Ferreira JJ, Rascol O (2022). COMT inhibitors in the management of Parkinson's disease. CNS Drugs..

[10] Jiménez-Jiménez FJ, Alonso-Navarro H, García-Martín E, Agúndez JA (2014). COMT gene and risk for Parkinson's disease: a systematic review and meta-analysis. Pharmacogenet. Genom..

[11] Won JH, Kim M, Youn J, Park H (2020). Prediction of age at onset in Parkinson’s disease using objective specific neuroimaging genetics based on a sparse canonical correlation analysis. Sci. Rep..

[12] Lee A, Qiu A (2016). Modulative effects of COMT haplotype on age-related associations with brain morphology. Hum. Brain Mapp..

[13] Salat D, Noyce AJ, Schrag A, Tolosa E (2016). Challenges of modifying disease progression in prediagnostic Parkinson's disease. Lancet Neurol..

[14] Marek K, Jennings D, Lasch S, Siderowf A, Tanner C, Simuni T (2011). The Parkinson progression marker initiative (PPMI). Progress in neurobiology..

[15] Matloff W, Toga A. Allelic status of selected Parkinson disease-associated variants for PPMI subjects with available whole-genome sequencing data. (2011).

[16] Slatkin M (2008). Linkage disequilibrium–understanding the evolutionary past and mapping the medical future. Nat. Rev. Genet..

[17] Rogers AR, Huff C (2009). Linkage disequilibrium between loci with unknown phase. Genetics..

[18] Miles A, Bot Pi, R M, Ralph P, Kelleher J, Schelker M, et al. cggh/scikit-allel: v1.3.7. v1.3.7 ed: Zenodo; (2023).

[19] Tang D, Chen M, Huang X, Zhang G, Zeng L, Zhang G (2023). SRplot: A free online platform for data visualization and graphing. PLoS One..

[20] Liao B, Li X, Cai L, Cao Z, Chen H (2015). A hierarchical clustering method of selecting kernel SNP to unify informative SNP and tag SNP. IEEE/ACM Trans. Comput. Biol. Bioinf..

[21] Reuter M, Schmansky NJ, Rosas HD, Fischl B (2012). Within-subject template estimation for unbiased longitudinal image analysis. NeuroImage..

[22] Reuter M, Fischl B (2011). Avoiding asymmetry-induced bias in longitudinal image processing. NeuroImage..

[23] Edwards LJ, Muller KE, Wolfinger RD, Qaqish BF, Schabenberger O (2008). An R2 statistic for fixed effects in the linear mixed model. Stat. Med..

[24] Nakagawa S, Johnson PCD, Schielzeth H (2017). The coefficient of determination R(2) and intra-class correlation coefficient from generalized linear mixed-effects models revisited and expanded. J. R Soc. Interface..

[25] Bates D, Mächler M, Bolker B, Walker S (2015). Fitting linear mixed-effects models using lme4. J. Stat. Softw..

[26] Brzyski D, Peterson CB, Sobczyk P, Candès EJ, Bogdan M, Sabatti C (2017). Controlling the rate of GWAS false discoveries. Genetics..

[27] Brinster R, Köttgen A, Tayo BO, Schumacher M, Sekula P (2018). Control procedures and estimators of the false discovery rate and their application in low-dimensional settings: an empirical investigation. BMC Bioinf..

[28] Williams-Gray CH, Evans JR, Goris A, Foltynie T, Ban M, Robbins TW (2009). The distinct cognitive syndromes of Parkinson's disease: 5 year follow-up of the CamPaIGN cohort. Brain..

[29] Cheng H, Li W, Gan C, Zhang B, Jia Q, Wang K (2016). The COMT (rs165599) gene polymorphism contributes to chemotherapy-induced cognitive impairment in breast cancer patients. Am. J. Transl. Res..

[30] du Boisgueheneuc F, Levy R, Volle E, Seassau M, Duffau H, Kinkingnehun S (2006). Functions of the left superior frontal gyrus in humans: a lesion study. Brain..

[31] Deschamps I, Baum SR, Gracco VL (2014). On the role of the supramarginal gyrus in phonological processing and verbal working memory: evidence from rTMS studies. Neuropsychologia..

[32] Wada S, Honma M, Masaoka Y, Yoshida M, Koiwa N, Sugiyama H (2021). Volume of the right supramarginal gyrus is associated with a maintenance of emotion recognition ability. PLoS One..

[33] DiGuiseppi J, Tadi P. Neuroanatomy, Postcentral Gyrus. StatPearls. Treasure Island (FL) ineligible companies. Disclosure: Prasanna Tadi declares no relevant financial relationships with ineligible companies.: StatPearls Publishing Copyright © 2024, StatPearls Publishing LLC.; (2024).

[34] Servaas MN, Geerligs L, Bastiaansen JA, Renken RJ, Marsman J-BC, Nolte IM (2017). Associations between genetic risk, functional brain network organization and neuroticism. Brain Imag. Behav..

[35] Gozukara Bag HG (2018). Association between COMT gene rs165599 SNP and schizophrenia: A meta-analysis of case-control studies. Mol. Genet. Genomic Med..

[36] Jugurnauth SK, Chen CK, Barnes MR, Li T, Lin SK, Liu HC (2011). A COMT gene haplotype associated with methamphetamine abuse. Pharmacogenet. Genom..

[37] Matsuzaka CT, Christofolini D, Ota VK, Gadelha A, Berberian AA, Noto C (2017). Catechol-O-methyltransferase (COMT) polymorphisms modulate working memory in individuals with schizophrenia and healthy controls. Braz. J. Psychiatry..

